# 4,4′-Bipyridine–4-(*p*-toluene­sulfonamido)­benzoic acid (1/2)

**DOI:** 10.1107/S1600536811034544

**Published:** 2011-08-27

**Authors:** Miao-Ling Huang

**Affiliations:** a College of Chemistry and Life Sciences, Quanzhou Normal University, Fujian 362000, People’s Republic of China

## Abstract

In the title compound, C_14_H_13_NO_4_S·0.5C_10_H_8_N_2_, the two benzene rings are nearly perpendicular to each other [dihedral angle = 83.21 (10)°]. The bipyridine mol­ecule is centrosymmetric, the mid-point of the C—C bond linking the pyridine rings being located on an inversion center. Inter­molecular N—H⋯O and O—H⋯N hydrogen bonds and weak inter­molecular C—H⋯O hydrogen bonds are present in the crystal structure.

## Related literature

For the background to the compound, see: Antolini *et al.* (1984 ▶); Menabue & Saladini (1988 ▶).
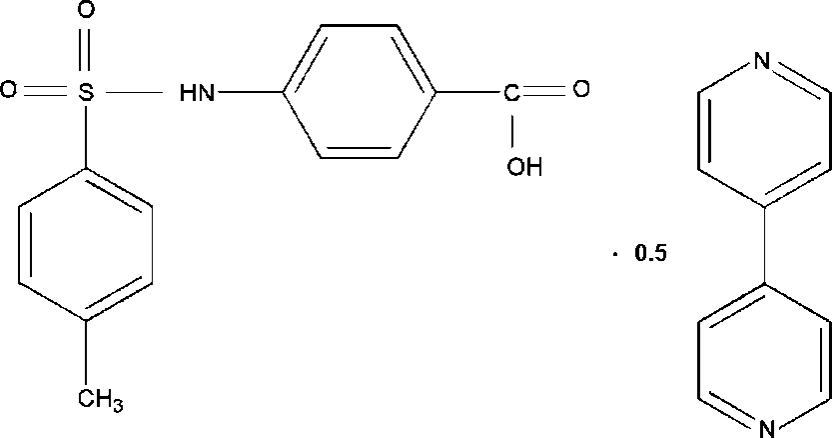


## Experimental

### 

#### Crystal data

C_14_H_13_NO_4_S·0.5C_10_H_8_N_2_
*M* *_r_* = 369.41
Monoclinic, 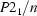

*a* = 5.8732 (7) Å
*b* = 8.124 (1) Å
*c* = 36.806 (5) Å
β = 94.137 (2)°
*V* = 1751.6 (4) Å^3^
*Z* = 4
Mo *K*α radiation
μ = 0.21 mm^−1^
*T* = 296 K
0.39 × 0.24 × 0.21 mm

#### Data collection

Bruker SMART CCD area-detector diffractometer
Absorption correction: multi-scan (*SADABS*; Bruker, 2001 ▶) *T*_min_ = 0.922, *T*_max_ = 0.957
7704 measured reflections
3234 independent reflections
2255 reflections with *I* > 2σ(*I*)
*R*_int_ = 0.026

#### Refinement

*R*[*F* ^2^ > 2σ(*F* ^2^)] = 0.039
*wR*(*F* ^2^) = 0.107
*S* = 0.99
3234 reflections
237 parameters
H-atom parameters constrained
Δρ_max_ = 0.21 e Å^−3^
Δρ_min_ = −0.24 e Å^−3^

### 

Data collection: *SMART* (Bruker, 2007 ▶); cell refinement: *SAINT* (Bruker, 2007 ▶); data reduction: *SAINT*; program(s) used to solve structure: *SHELXTL* (Sheldrick, 2008 ▶); program(s) used to refine structure: *SHELXTL*; molecular graphics: *SHELXTL*; software used to prepare material for publication: *SHELXTL*.

## Supplementary Material

Crystal structure: contains datablock(s) global, I. DOI: 10.1107/S1600536811034544/xu5301sup1.cif

Structure factors: contains datablock(s) I. DOI: 10.1107/S1600536811034544/xu5301Isup2.hkl

Supplementary material file. DOI: 10.1107/S1600536811034544/xu5301Isup3.cml

Additional supplementary materials:  crystallographic information; 3D view; checkCIF report

## Figures and Tables

**Table 1:** Hydrogen-bond geometry (Å, °)

*D*—H⋯*A*	*D*—H	H⋯*A*	*D*⋯*A*	*D*—H⋯*A*
N1—H1⋯O1^i^	0.86	2.03	2.861 (2)	162
O2—H2*A*⋯N2^ii^	0.82	1.87	2.691 (2)	175
C2—H2⋯O4^ii^	0.93	2.51	3.413 (2)	163

Symmetry codes: (i) 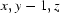; (ii) 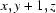.

**Fig. 1.**
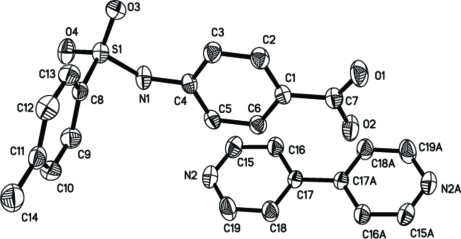
The ORTEP drawing of the title compound (I). Displacement ellipsoids are drawn at 30% probability level. All hydrogen atams have been omitted for reasons of clarity.

**Fig. 2.**
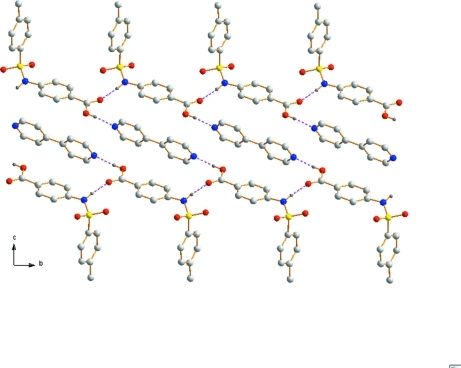
A view of the hydrogen bonds (dotted lines) in the crystal structure of the title compound (I).

**Fig. 3.**
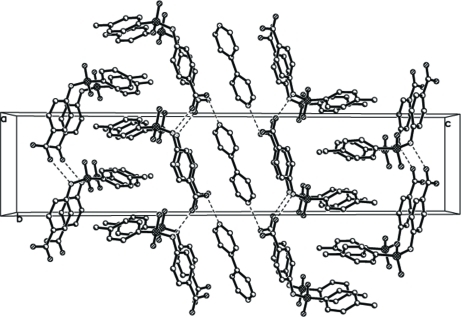
The crystal packing of the title compound (I), viewed along the c axis.

## supplementary crystallographic information

### Comment

N-Protected amino acids possess *R*-CONH-*R*' group analogous to the structure of O-terminal of peptide and proteins (Menabue & Saladini, 1988, Antolini *et al.*, 1984). The substitution of an Ar—SO_2_-group on amine makes the 4-aminobenzeoic acid increase the coordination donors to three types-O, N donors from carboxyl, sulfoxyl and amine respectively, which may result in different coordination mode. In this paper, we attempt synthesizing the *N*-*p*-tolysulfonyl-4-amionbenzoic acid adduct of Erbium and 4,4'-bipyridine, but the result to get the title compound.

The title compound contains of one *N*-*p*-tolysulfonyl-4-amionbenzoic acid molecule and one 4,4'-bipyridine in the asymmetric unit (Fig.1). The molecule has a C4—N1—S1—C8 of 74.247 (2) °, and the dihedral angle between the benzene rings is 83.213 (6) °. There exit intermolecular hydrogen bonds between carboxylate group oxygen atoms, secondary amine nitrogen atoms and pyridine ring nitrogen atoms of N—H···O and O—H···N. Then, an extended one-dimensional chain structure along *b* axis is formed (Fig.2). It is interesting that the hydrogen bonds play an important role in forming the one-dimensional structure and stabilize the superamolecular structure(Fig.3).

### Experimental

A mixture of *N*-*p*-tolysulfonylchloride (1 mmol) and 4-amionbenzoic acid (1 mmol) in water (20 mL) was stirred at room temperature for 10 h. Then HCl (12 mol/*L*) was slowly added to the resulting solution. The mixture was stirred for 5 min and filtrated. The precipitate was washed by distilled water, and dried to constant heavy [product 1].

To a solution of the product 1 (1 mmol) in water-DMF 1:1 (10 mL), an aqueous solution (5 ml) of Er(NO_3_)_3_.6H_2_O (0.5 mmol) and a solution of 4,4'-bipyridine (0.25 mmol) in ethanol (95%, 5 ml) was added. After refluxing for 12 h at 343 K, the mixture was filtered off while hot. The block colourless single crystals suitable for X-ray analysis were obtained by slow evaporation of the filtrate at room temperature after one week.

### Refinement

H atoms were placed in calculated positions and treated as riding on their parent atoms (C—H = 0.93–0.96 Å, N—H = 0.86 Å, O—H = 0.82 Å) and U_iso_(H) = 1.2U_eq_(C,N) and 1.5U_eq_(O).

### Crystal data

**Table:**

C_14_H_13_NO_4_S·0.5C_10_H_8_N_2_	*F*(000) = 772
*M**_r_* = 369.41	*D*_x_ = 1.401 Mg m^−^^3^
Monoclinic, *P*2_1_/*n*	Mo *K*α radiation, λ = 0.71073 Å
Hall symbol: -P 2yn	Cell parameters from 2422 reflections
*a* = 5.8732 (7) Å	θ = 2.6–23.6°
*b* = 8.124 (1) Å	µ = 0.21 mm^−^^1^
*c* = 36.806 (5) Å	*T* = 296 K
β = 94.137 (2)°	Block, colourless
*V* = 1751.6 (4) Å^3^	0.39 × 0.24 × 0.21 mm
*Z* = 4	

### Data collection

**Table:**

Bruker SMART CCD area-detector diffractometer	3234 independent reflections
Radiation source: fine-focus sealed tube	2255 reflections with *I* > 2σ(*I*)
graphite	*R*_int_ = 0.026
φ and ω scans	θ_max_ = 25.5°, θ_min_ = 2.6°
Absorption correction: multi-scan (*SADABS*; Bruker, 2001)	*h* = −6→7
*T*_min_ = 0.922, *T*_max_ = 0.957	*k* = −9→7
7704 measured reflections	*l* = −44→44

### Refinement

**Table:**

Refinement on *F*^2^	Secondary atom site location: difference Fourier map
Least-squares matrix: full	Hydrogen site location: inferred from neighbouring sites
*R*[*F*^2^ > 2σ(*F*^2^)] = 0.039	H-atom parameters constrained
*wR*(*F*^2^) = 0.107	*w* = 1/[σ^2^(*F*_o_^2^) + (0.0567*P*)^2^] where *P* = (*F*_o_^2^ + 2*F*_c_^2^)/3
*S* = 0.99	(Δ/σ)_max_ < 0.001
3234 reflections	Δρ_max_ = 0.21 e Å^−^^3^
237 parameters	Δρ_min_ = −0.24 e Å^−^^3^
0 restraints	Extinction correction: *SHELXTL* (Sheldrick, 2008), Fc^*^=kFc[1+0.001xFc^2^λ^3^/sin(2θ)]^-1/4^
Primary atom site location: structure-invariant direct methods	Extinction coefficient: 0.0079 (11)

### Special details

**Table:**

Experimental. IR(KBr): 3439(*s*), 3214(*versus*), 2493(w), 1922(w), 1668(*s*), 1603(*versus*), 1511 (w), 1477(vw), 1409(*m*), 1341(*s*), 1314(*s*), 1289(*s*), 1232(*m*), 1216(*m*), 1158(*versus*), 1092(*versus*), 1004(*m*), 923(*m*), 860(*m*), 803(*m*), 779(*m*), 699(*m*), 668(*m*), 626(*s*), 574(*s*), 548(*s*), 521(*s*), 502(*m*)cm^-1^.
Geometry. All e.s.d.'s (except the e.s.d. in the dihedral angle between two l.s. planes) are estimated using the full covariance matrix. The cell e.s.d.'s are taken into account individually in the estimation of e.s.d.'s in distances, angles and torsion angles; correlations between e.s.d.'s in cell parameters are only used when they are defined by crystal symmetry. An approximate (isotropic) treatment of cell e.s.d.'s is used for estimating e.s.d.'s involving l.s. planes.
Refinement. Refinement of *F*^2^ against ALL reflections. The weighted *R*-factor *wR* and goodness of fit *S* are based on *F*^2^, conventional *R*-factors *R* are based on *F*, with *F* set to zero for negative *F*^2^. The threshold expression of *F*^2^ > σ(*F*^2^) is used only for calculating *R*-factors(gt) *etc*. and is not relevant to the choice of reflections for refinement. *R*-factors based on *F*^2^ are statistically about twice as large as those based on *F*, and *R*- factors based on ALL data will be even larger.

### Fractional atomic coordinates and isotropic or equivalent isotropic displacement parameters (Å^2^)

**Table:**

	*x*	*y*	*z*	*U*_iso_*/*U*_eq_	Occ. (<1)
C1	0.5273 (3)	1.1613 (2)	0.09831 (5)	0.0455 (5)	
C2	0.3358 (4)	1.1518 (2)	0.11792 (6)	0.0572 (6)	
H2	0.2583	1.2478	0.1232	0.069*	
C3	0.2574 (4)	1.0027 (3)	0.12985 (6)	0.0571 (6)	
H3	0.1270	0.9983	0.1427	0.069*	
C4	0.3736 (3)	0.8592 (2)	0.12256 (5)	0.0450 (5)	
C5	0.5699 (3)	0.8680 (2)	0.10403 (5)	0.0495 (5)	
H5	0.6516	0.7727	0.0998	0.059*	
C6	0.6449 (3)	1.0172 (2)	0.09175 (5)	0.0495 (5)	
H6	0.7756	1.0215	0.0789	0.059*	
C7	0.5983 (4)	1.3224 (3)	0.08396 (6)	0.0544 (5)	
C8	0.3248 (3)	0.6500 (2)	0.20402 (5)	0.0432 (5)	
C9	0.5294 (3)	0.5665 (2)	0.20487 (6)	0.0569 (6)	
H9	0.5745	0.5147	0.1840	0.068*	
C10	0.6658 (4)	0.5605 (3)	0.23665 (7)	0.0622 (6)	
H10	0.8045	0.5052	0.2370	0.075*	
C11	0.6024 (4)	0.6350 (3)	0.26843 (6)	0.0558 (6)	
C12	0.3979 (4)	0.7180 (3)	0.26679 (6)	0.0606 (6)	
H12	0.3521	0.7696	0.2876	0.073*	
C13	0.2597 (3)	0.7264 (2)	0.23507 (6)	0.0541 (5)	
H13	0.1223	0.7836	0.2346	0.065*	
C14	0.7556 (4)	0.6262 (3)	0.30290 (7)	0.0854 (8)	
H14A	0.8893	0.5632	0.2986	0.128*	0.50
H14B	0.6755	0.5743	0.3217	0.128*	0.50
H14C	0.7997	0.7354	0.3105	0.128*	0.50
H14D	0.6870	0.6854	0.3219	0.128*	0.50
H14E	0.9008	0.6743	0.2988	0.128*	0.50
H14F	0.7766	0.5132	0.3100	0.128*	0.50
C15	0.7243 (5)	0.6866 (3)	0.00908 (8)	0.0912 (9)	
H15	0.5864	0.6378	0.0011	0.109*	
C16	0.7715 (4)	0.8401 (3)	−0.00428 (7)	0.0784 (8)	
H16	0.6671	0.8914	−0.0208	0.094*	
C17	0.9707 (3)	0.9168 (2)	0.00662 (5)	0.0447 (5)	
C18	1.1119 (4)	0.8311 (3)	0.03098 (6)	0.0745 (7)	
H18	1.2501	0.8772	0.0397	0.089*	
C19	1.0513 (5)	0.6771 (3)	0.04270 (7)	0.0818 (8)	
H19	1.1528	0.6220	0.0590	0.098*	
N1	0.2922 (3)	0.70046 (19)	0.13109 (4)	0.0559 (5)	
H1	0.3281	0.6224	0.1168	0.067*	
N2	0.8605 (4)	0.6044 (2)	0.03226 (5)	0.0681 (5)	
O1	0.5113 (3)	1.45211 (18)	0.09093 (5)	0.0797 (5)	
O2	0.7586 (3)	1.31137 (17)	0.06109 (5)	0.0780 (5)	
H2A	0.7877	1.4033	0.0535	0.117*	
O3	−0.0355 (2)	0.76526 (18)	0.16802 (4)	0.0655 (4)	
O4	0.0804 (3)	0.47892 (17)	0.15660 (4)	0.0686 (5)	
S1	0.14014 (9)	0.64661 (6)	0.164306 (13)	0.05199 (19)	

### Atomic displacement parameters (Å^2^)

**Table:**

	*U*^11^	*U*^22^	*U*^33^	*U*^12^	*U*^13^	*U*^23^
C1	0.0551 (12)	0.0388 (12)	0.0428 (10)	0.0030 (9)	0.0042 (9)	0.0038 (9)
C2	0.0691 (14)	0.0414 (13)	0.0628 (13)	0.0050 (11)	0.0161 (11)	−0.0028 (10)
C3	0.0639 (14)	0.0463 (13)	0.0638 (14)	0.0018 (11)	0.0220 (11)	0.0019 (10)
C4	0.0599 (12)	0.0394 (11)	0.0357 (10)	0.0014 (10)	0.0032 (9)	0.0032 (8)
C5	0.0593 (12)	0.0385 (12)	0.0507 (11)	0.0077 (10)	0.0053 (10)	0.0044 (9)
C6	0.0541 (12)	0.0481 (13)	0.0470 (11)	0.0033 (10)	0.0093 (9)	0.0035 (9)
C7	0.0638 (14)	0.0444 (13)	0.0556 (13)	−0.0003 (11)	0.0078 (11)	0.0016 (10)
C8	0.0475 (11)	0.0334 (10)	0.0504 (11)	0.0002 (9)	0.0148 (9)	0.0041 (9)
C9	0.0587 (13)	0.0550 (14)	0.0591 (13)	0.0085 (11)	0.0190 (11)	−0.0022 (10)
C10	0.0499 (13)	0.0563 (14)	0.0808 (17)	0.0067 (11)	0.0077 (12)	0.0102 (12)
C11	0.0587 (13)	0.0477 (13)	0.0606 (13)	−0.0144 (11)	0.0025 (10)	0.0115 (11)
C12	0.0684 (14)	0.0596 (14)	0.0551 (13)	−0.0068 (12)	0.0126 (11)	−0.0109 (11)
C13	0.0549 (12)	0.0498 (13)	0.0591 (13)	0.0062 (10)	0.0140 (10)	−0.0053 (10)
C14	0.0839 (17)	0.092 (2)	0.0770 (17)	−0.0257 (15)	−0.0137 (14)	0.0242 (14)
C15	0.0822 (19)	0.0621 (17)	0.127 (2)	−0.0281 (14)	−0.0092 (17)	0.0158 (16)
C16	0.0726 (16)	0.0589 (16)	0.0998 (19)	−0.0182 (13)	−0.0212 (14)	0.0224 (13)
C17	0.0542 (12)	0.0437 (11)	0.0364 (10)	−0.0062 (10)	0.0049 (9)	−0.0006 (8)
C18	0.0802 (16)	0.0666 (16)	0.0721 (15)	−0.0210 (13)	−0.0252 (13)	0.0218 (12)
C19	0.110 (2)	0.0661 (17)	0.0666 (16)	−0.0104 (16)	−0.0159 (15)	0.0225 (13)
N1	0.0848 (12)	0.0381 (10)	0.0466 (9)	−0.0041 (9)	0.0182 (9)	0.0006 (7)
N2	0.0893 (15)	0.0513 (12)	0.0658 (12)	−0.0105 (11)	0.0208 (11)	0.0070 (10)
O1	0.1032 (13)	0.0362 (9)	0.1041 (13)	0.0068 (9)	0.0379 (10)	0.0035 (8)
O2	0.1009 (13)	0.0472 (9)	0.0918 (12)	0.0005 (9)	0.0475 (10)	0.0116 (9)
O3	0.0551 (8)	0.0692 (10)	0.0733 (10)	0.0123 (8)	0.0112 (7)	0.0181 (8)
O4	0.0911 (11)	0.0506 (9)	0.0640 (9)	−0.0236 (8)	0.0055 (8)	0.0020 (7)
S1	0.0603 (3)	0.0455 (3)	0.0507 (3)	−0.0042 (3)	0.0078 (2)	0.0067 (2)

### Geometric parameters (Å, °)

**Table:**

C1—C2	1.382 (3)	C12—H12	0.9300
C1—C6	1.389 (2)	C13—H13	0.9300
C1—C7	1.482 (3)	C14—H14A	0.9600
C2—C3	1.379 (3)	C14—H14B	0.9600
C2—H2	0.9300	C14—H14C	0.9600
C3—C4	1.387 (3)	C14—H14D	0.9600
C3—H3	0.9300	C14—H14E	0.9600
C4—C5	1.383 (3)	C14—H14F	0.9600
C4—N1	1.419 (2)	C15—N2	1.309 (3)
C5—C6	1.377 (3)	C15—C16	1.376 (3)
C5—H5	0.9300	C15—H15	0.9300
C6—H6	0.9300	C16—C17	1.360 (3)
C7—O1	1.207 (2)	C16—H16	0.9300
C7—O2	1.310 (2)	C17—C18	1.367 (3)
C8—C9	1.378 (3)	C17—C17^i^	1.485 (3)
C8—C13	1.379 (3)	C18—C19	1.379 (3)
C8—S1	1.7563 (19)	C18—H18	0.9300
C9—C10	1.370 (3)	C19—N2	1.300 (3)
C9—H9	0.9300	C19—H19	0.9300
C10—C11	1.391 (3)	N1—S1	1.6253 (16)
C10—H10	0.9300	N1—H1	0.8600
C11—C12	1.375 (3)	O2—H2A	0.8200
C11—C14	1.503 (3)	O3—S1	1.4254 (14)
C12—C13	1.375 (3)	O4—S1	1.4300 (14)
			
C2—C1—C6	118.52 (18)	H14A—C14—H14C	109.5
C2—C1—C7	119.73 (18)	H14B—C14—H14C	109.5
C6—C1—C7	121.73 (18)	C11—C14—H14D	109.5
C3—C2—C1	121.22 (19)	H14A—C14—H14D	141.1
C3—C2—H2	119.4	H14B—C14—H14D	56.3
C1—C2—H2	119.4	H14C—C14—H14D	56.3
C2—C3—C4	119.8 (2)	C11—C14—H14E	109.5
C2—C3—H3	120.1	H14A—C14—H14E	56.3
C4—C3—H3	120.1	H14B—C14—H14E	141.1
C5—C4—C3	119.45 (18)	H14C—C14—H14E	56.3
C5—C4—N1	117.54 (17)	H14D—C14—H14E	109.5
C3—C4—N1	122.87 (19)	C11—C14—H14F	109.5
C6—C5—C4	120.33 (18)	H14A—C14—H14F	56.3
C6—C5—H5	119.8	H14B—C14—H14F	56.3
C4—C5—H5	119.8	H14C—C14—H14F	141.1
C5—C6—C1	120.66 (19)	H14D—C14—H14F	109.5
C5—C6—H6	119.7	H14E—C14—H14F	109.5
C1—C6—H6	119.7	N2—C15—C16	124.4 (2)
O1—C7—O2	122.1 (2)	N2—C15—H15	117.8
O1—C7—C1	124.1 (2)	C16—C15—H15	117.8
O2—C7—C1	113.71 (18)	C17—C16—C15	120.1 (2)
C9—C8—C13	119.75 (19)	C17—C16—H16	120.0
C9—C8—S1	119.75 (15)	C15—C16—H16	120.0
C13—C8—S1	120.35 (15)	C16—C17—C18	115.33 (19)
C10—C9—C8	119.48 (19)	C16—C17—C17^i^	122.4 (2)
C10—C9—H9	120.3	C18—C17—C17^i^	122.3 (2)
C8—C9—H9	120.3	C17—C18—C19	120.6 (2)
C9—C10—C11	121.8 (2)	C17—C18—H18	119.7
C9—C10—H10	119.1	C19—C18—H18	119.7
C11—C10—H10	119.1	N2—C19—C18	123.8 (2)
C12—C11—C10	117.5 (2)	N2—C19—H19	118.1
C12—C11—C14	121.9 (2)	C18—C19—H19	118.1
C10—C11—C14	120.6 (2)	C4—N1—S1	128.58 (14)
C11—C12—C13	121.5 (2)	C4—N1—H1	115.7
C11—C12—H12	119.3	S1—N1—H1	115.7
C13—C12—H12	119.3	C19—N2—C15	115.7 (2)
C12—C13—C8	119.96 (19)	C7—O2—H2A	109.5
C12—C13—H13	120.0	O3—S1—O4	119.64 (10)
C8—C13—H13	120.0	O3—S1—N1	109.31 (9)
C11—C14—H14A	109.5	O4—S1—N1	104.32 (9)
C11—C14—H14B	109.5	O3—S1—C8	108.50 (9)
H14A—C14—H14B	109.5	O4—S1—C8	107.93 (9)
C11—C14—H14C	109.5	N1—S1—C8	106.38 (9)
			
C6—C1—C2—C3	−2.1 (3)	C9—C8—C13—C12	−0.5 (3)
C7—C1—C2—C3	176.19 (18)	S1—C8—C13—C12	175.18 (15)
C1—C2—C3—C4	0.9 (3)	N2—C15—C16—C17	−0.2 (5)
C2—C3—C4—C5	1.3 (3)	C15—C16—C17—C18	−0.2 (4)
C2—C3—C4—N1	−174.24 (18)	C15—C16—C17—C17^i^	−179.4 (3)
C3—C4—C5—C6	−2.3 (3)	C16—C17—C18—C19	0.7 (4)
N1—C4—C5—C6	173.49 (16)	C17^i^—C17—C18—C19	179.9 (2)
C4—C5—C6—C1	1.1 (3)	C17—C18—C19—N2	−0.9 (4)
C2—C1—C6—C5	1.1 (3)	C5—C4—N1—S1	153.34 (15)
C7—C1—C6—C5	−177.15 (18)	C3—C4—N1—S1	−31.0 (3)
C2—C1—C7—O1	7.0 (3)	C18—C19—N2—C15	0.5 (4)
C6—C1—C7—O1	−174.8 (2)	C16—C15—N2—C19	0.1 (4)
C2—C1—C7—O2	−169.59 (19)	C4—N1—S1—O3	42.72 (19)
C6—C1—C7—O2	8.6 (3)	C4—N1—S1—O4	171.78 (16)
C13—C8—C9—C10	0.0 (3)	C4—N1—S1—C8	−74.25 (18)
S1—C8—C9—C10	−175.72 (15)	C9—C8—S1—O3	−169.22 (15)
C8—C9—C10—C11	0.8 (3)	C13—C8—S1—O3	15.07 (18)
C9—C10—C11—C12	−1.0 (3)	C9—C8—S1—O4	59.76 (17)
C9—C10—C11—C14	179.9 (2)	C13—C8—S1—O4	−115.95 (17)
C10—C11—C12—C13	0.5 (3)	C9—C8—S1—N1	−51.71 (17)
C14—C11—C12—C13	179.54 (19)	C13—C8—S1—N1	132.58 (16)
C11—C12—C13—C8	0.3 (3)		

Symmetry codes: (i) −*x*+2, −*y*+2, −*z*.

### Hydrogen-bond geometry (Å, °)

**Table:**

*D*—H···*A*	*D*—H	H···*A*	*D*···*A*	*D*—H···*A*
N1—H1···O1^ii^	0.86	2.03	2.861 (2)	162
O2—H2A···N2^iii^	0.82	1.87	2.691 (2)	175
C2—H2···O4^iii^	0.93	2.51	3.413 (2)	163

Symmetry codes: (ii) *x*, *y*−1, *z*; (iii) *x*, *y*+1, *z*.
